# Further Remarks on Isotonic Sea Water in Therapeutics

**Published:** 1913-11

**Authors:** Rollin H. Stevens

**Affiliations:** Clinical Professor of Dermatology, Detroit College of Medicine, Dermatologist to Grace Hospital, German Policlinic, Etc.


### Further Remarks on Isotonic Sea Water in Therapeutics.

BY ROLLIN H. STEVENS, M.D.
Clinical Professor of Dermatology, Detroit College of Medicine,
Dermatologist to Grace Hospital, German Policlinic, Etc.

IN my article which appeared in the BUFFALO MEDICAL JOURNAL in February of this year, I briefly described Quinton's theories of the origin of the animal plasma from the sea, and the preservation of the chemical and physical characters of the latter in the plasma throughout the animal series.

This is briefly stated in Quinton's more general law of Marine Constancy, thus:

"Animal life, which appeared in the cellular state in the seas, has tended to maintain for its high cellular functioning, throughout the zoological series, the cells composing each organism in a marine medium. It has not maintained this medium in all organisms, but those in which the maintenance has not been effected have undergone a vital decadence."

The latter clause applies only to a comparatively few aerial and fresh water invertebrates, all the vertebrates conforming to this law. He goes further and states that for the high functioning of cells, as in the case of mammalia, birds, and some insects, the original temperature and saline concentration has also been preserved, the sea having originally in the precambrian period probably been about 37° C+ and more dilute than at present.

As a part of the basis for the conclusions he compares analyses of modern sea waters, mineral waters (from the beds of ancient seas) and of plasmas of various forms of animal life. He finds the mineral constitution of plasmas of higher vertebrates, for instance, and of ancient or modern sea water to bear a very close resemblance—the difference between ancient and modern sea waters being one chiefly of concentration.

For instance, the elements found in sea water have been arranged in groups according to their importance, as follows:

1. Cl., and Na. composing .84 of total solids.

2. S. Mg. K. and Ca composing .14 of total solids.

3. Br. C, Si, Fe, N, Fol., P, Li, I, Bo. composing 0.089997 of total solids.

4. As., Cu. Ag., Au., Zn., Mn., Str., Ba., Cocsium, Rubid, Al., Pb. and Co., forming together 0.000003.

Quinton divides the body into four great divisions, namely:

1. "Vital Medium"—the total plasma composing 1/3 of the weight of the body and bathing all the cells.

2. Living material—composed of all the cells of the organism endowed with life.

3. Dead material—or the ensemble of cellular productions only later playing in the organism the purely physical role of union, isolation, protection, or support.

4. Secreting (or excreting) material—all the cellular secretions for organic needs.

By "vital medium," or plasma, Quinton means the fluid portion of the entire body, not only that of the blood, and he values its quantity at 1/3 the weight of the body. The mass of blood is 1/12 the weight of the body, and is composed of two almost equal portions—the plasma and cells. The rest of the plasma comprises that of imbibition in the various cells (devoid of vessels) the interstitial and the lymphatic. Quinton shows by physiological experiment and chemical analyses that the former penetrates the fundamental substance of epithelium, connective tissue, and cartilage.

Vital Medium or Plasma—Examinations of plasma show Cl. and Na. domination in almost equal proportions, constituting .90 of total solids.

In the second group of plasma elements we find K, Ca, Mg, together forming .07 or .08 of the dissolved salts.

In the third group are P. C. Si. N (ammonia) Fl. Fe., I, Br., Mn. Cu. Pb. Zn. Li. Ag. As. Bor. Ba. Al., constituting the balance.

These elements do not appear in exactly the same proportions as in modern sea water, but due allowance must be made for greater concentration having taken place in the ages since the ancient seas, also for changes in the animal economy due to environment and the many vicissitudes of life in the same time.

But here in plasma are enumerated only twenty-four elements as against twenty-nine in sea water. Of the five marine bodies not yet recognized in the animal organism, three, strontium, rubidium and coesium, Quinton says, are more than probably present. Gold is also likely there. There is no information concerning the presence of cobalt alone to complete the group. But Quinton points out that the sole analysis which determined cobalt present in sea water was a physiological one, for it is in organic combination. So we find practically the same elements in approximately the same doses in plasma as in sea water. Many of the elements—those of the latter group—are in extraordinarily infinitesimal amounts, and yet, according to a physiological microchemistry now fairly well under way, these infinitesimal elements evidently have an important role to play in the organism in these doses, and in these doses alone.

The importance of an element in the organism is not to be gauged directly with the amount of it present. We know that loss of the small amount of iodine present means death. Hence its importance is as great as salt. Other elements will probably be discovered in both sea water and animal plasma. Indeed, there is much evidence, though Quinton does not mention it, to show that radium is present in both. So the close similiarity of sea water and animal plasma is very striking, both as to their qualitative and their quantitative chemical composition.

*Living Material.*—In this group is included all the cells of the organism endowed with life—epithelial, glandular, amœboid, cartilaginous osseous, muscular, nervous, blood, and all the cells of different types according to the vital functions which they perform. Some are bathed directly in the lymph stream—the blood cells for instance—while others which are massed without blood vessels—the epithelia for instance—are bathed indirectly, only through the intermediary of the uniting substance, which is impregnated with the plasma.

The mineral composition of this material is quite typical and quite different from that of the "vital medium" or plasma.

The dominating salt of the latter and of sea water is chloride of sodium, but the principal salt of "living material" is the phosphate of potassium in the proportion of about .64 or the total salts.

*"Dead, or not Immediately Living Material."*

By this term Quinton means all the solid elements of the organism which are not living cells. Some of this material is cellular, as the corneal layer of the epidermis, the nails, the hair, etc., and other is extra-cellular, as the fundamental substances (stroma) of mucous tissue, cartilage, bone, fiebres of elastic and fibrous tissue, enamel of teeth, intercellular cement, etc., etc.

The mineral composition of this material differs again from the other two, and analyses by many different observers show it to be composed chiefly of phosphate of lime in the vertebrates, in the proportion about .90 of total salts; and of carbonate of lime in vertebrates in the proportion of about .65 to .99 of total salts.

*Secreting Material.*—This material is of numerous kinds in the organism and is composed of the material secreted by many closed glands into the vital medium or plasma, where it remains largely unknown and undifferentiated; the material secreted for digestive purposes, such as gastric juice, bile, pancreatic fluid, saliva, etc., excrementary material such as urine, sweat, etc., and nutritive material, as milk.

Analyses of secretive material show it to be very diverse; first, in the same animal according to glandular function; second, diverse one animal from another for the same function; third, diverse in the same animal for the same function. So there is no mineral for secreting material that is typical or characteristic, thus differing from the saline constancy of the other three divisions.

There is no sample of secreted material which has the same mineral composition as the plasma or "vital medium."

Therefore, he concludes, the four kinds of material of which the body is composed present mineral personalities entirely distinct one from the other, and only one, the vital medium, is the same as sea water. "As these different personalities are constituted at the expense of a common alimentation, alimentation is then incapable of explaining any of them in their peculiar particularities. None is the passive result of alimentation."

Indeed, Quinton shows by a long series of chemical analyses of food stuffs of animals that the marine composition of the plasma is not only not realized *because* of alimentation, but *in spite* of alimentation, vegetable food stuffs and even exsanguinated meat, for instance, being actually poor in sodium chloride.

So Quinton justifies the marine origin of the plasma, and its perpetuation throughout the zoological series in practically the same original conditions of chemical composition, osmotic pressure, and temperature. This is so in at least those cases where there is the highest functioning of cells. If these conditions for any reason are not maintained, disease, decadence or death probably result.

We know so little about the nature of the various protective bodies, is it not possible that they are elementary, and consequently many of them exist only in the infinitesimal state—in too small quantities to be easily determined? If so, the beneficial result from the therapeutic use of sea water may be accounted for by the fact that some of these elements are thus supplied in assimilable form. However that may be, it has been demonstrated in thousands of cases in France that subcutaneous injections of sea water in many diverse kinds of disease are often followed by satisfactory improvements and cures. My own experience extending over nearly five years has amply confirmed these results.

Quinton's comparison of the body to a tube of culture media with micro-organisms growing thereon is so apt it will bear repetition. The body's envelope, skin and mucous membrance, is the tube or flask, the "vital medium," or plasma (representing 1/3 of the body) is the culture media, and growing therein are all the cells of the organism. In growing micro-organisms how important it is to have the culture media contain the proper organic and inorganic matter for their development. So if the culture medium for our cells for any reason is deficient in certain minerals, why not supply some normal culture medium by injecting some of the pure original into the circulation? This may not, and does not cure all ills. No such claim is made. Disease is due in the first place to lack of resistance, or insufficient protection; in the second place to infection with micro-organisms of one or more species. Sometimes building up resistance is all that is necessary, as in the treatment of tuberculosis by open air and feeding. The organisms in those cases can successfully withstand the ravages of the disease germs and overcome them. In other cases, syphilis, for instance, it seems to be necessary to give a bactericidal treatment in addition to strengthening resistance.

Theoretically sea water should build up resistance, and in several diseases it seems to do this so successfully that no other treatment is necessary.

In the class of diseases requiring also bactericidal treatment, especially chronic cases of tuberculosis and syphilis, sea water is a valuable adjuvant. I find it of some value in many skin diseases of a chronic nature, but particularly in psoriasis, where in large doses it seems to be almost a specific. I cannot report fully on these cases as yet, as time enough has not elapsed since I began to use the larger doses which have given much more uniform and satisfactory results than the smaller doses.

Recently in a case of general psoriasis of several years standing in a girl of sixteen, weighing about 105, the eruption began to fade after the first dose, and absolutely disappeared, leaving not a trace on the skin anywhere after ten doses of 300 to 400 c.c. each given a week apart.

In another case a man 32 years of age and weighing about 165, 400 c.c. doses once a week, improved, but did not make much headway. I increased to 600 c.c. and 700 c.c. at a dose and marked improvement was immediate. The case has only had two doses the latter size and is not yet healed.

Similar experience was had in an inveterate case in a woman 45 years of age and weighing 160. Only slight improvement followed doses of 50 c.c. to 200 c.c. twice a week and finally 400 c.c. once a week. But when 600 to 800 c.c. were given the eruption rapidly healed and a cure at least of a year's duration up to the present time was the result.

In all I have eight cases of psoriasis who have been faithful in taking the treatment. Seven of these have been influenced very favorably or apparently cured, while one, the first one treated—with small doses for several months—failed to respond. I believe now the trouble was due to insufficient dosage. There was a time when I dreaded seeing cases of psoriasis, because, as a rule, it was not possible to give more than temporary relief, with X-ray or ointments, but now I am hopeful of permanent cures. The dosage I find to be a very important matter. It will not do to give these doses to all cases. They aggravate some cases of eczema. For instance, a case of eczematous dermatitis involving the greater portion of the body could not bear more than 30 c.c. at a dose without decidedly unpleasant aggravations. This happened five times successively after doses of from 200 c.c. graded down finally to 30 c.c., which was the largest tolerant dose. The patient was a man 45 years of age and weighed about 120 lbs. I have noted similar aggravations in other cases of eczema, as well as in some other diseases. The aggravation was usually followed by slow improvement after an interval of a week or ten days.

Quinton states in his book the dose should be 100th to 150th of the body weight. That is, a man weighing 150 lbs. should receive a pint at a dose repeated once a week to ten days. However, this plan is not generally carried out in the dispensaries of Paris, 30 c.c. to 200 c.c. being the average doses given there.

There may be more or less severe reactions after injections of moderate or large sized doses. These reactions usually appear about four hours after, and take the form of fullness and aching of the part injected, headache, chill, fever, aching of bones—like the grippe, nausea. Any one or all of these symptoms may be present. Rarely they begin much earlier—immediately after or even during the injection. They pass off in from an hour or two to forty-eight hours. Slight nausea or disgust for food often is the only reaction and may persist a day or two.

In my experience no serious results have ever followed an injection, and they give the same report in the dispensaries of Paris, so the use of properly filtered isotonic sea water may be regarded as harmless.

The longer we use the therapeutic method, the more we are convinced it is a valuable adjunct to our therapeutic armamentarium in many chronic diseases. And we are beginning to regard it almost as a specific for psoriasis, but much further experience will be necessary, of course, before that can be proven.

---

* The fresh sea water is diluted in proportion of three parts of sea water to five parts of the purest distilled water, filtered thru filter paper, then twice thru germ proof (Chamberland) filters under suction into sterile containers, sealed up and kept in a cool, preferably cold place for use. For most work, sea water more than sixty days old should not be used. The older sea water however seems to work well in psoriasis.

